# The Image of Disease in Religious, Medical–Astrological and Social Discourses: Old Polish Literature as an Example of Early Modern European Mentality

**DOI:** 10.1007/s10943-020-01056-x

**Published:** 2020-06-24

**Authors:** Małgorzata Krzysztofik

**Affiliations:** Jan Kochanowski University in Kielce, ul. Uniwersytecka 17, Kielce, Poland

**Keywords:** Medical history, Religious discourse, Astrology, Aetiology of the disease, Social discourse

## Abstract

Today, the world is struggling with a coronavirus epidemic. People explain differently the causes and sense of this disease. Old Polish literature about diseases is representative for European thought in the modern era. The problem of the disease appears in old Polish literature in various discourses. The three most important are religious, medical–astrological and social discourse. In this article, I discuss basic paradigms of thinking connected with these discourses and the relationship between them. In the religious discourse, it is God who decides about health and illness. The pathological state of the organism can be both a trial and a punishment for the sinner. The medical and astrological discourse is based on ancient medicine, medieval medicine and astrology. It assumes a close dependence of human health on the balance of the fluids in the body and on the planetary system. The social discourse is dominated by epidemics of infectious diseases. It is a collection of advices for organizing a society during a pandemic.

## Introduction

The phenomenon of disease in each culture is the resultant of the level of knowledge, geographical location, understanding of philosophical schools, religiousness, social and political relations, economic factors as well as the competence and experience of physicians. Nowadays, disease processes are explained in the spirit of the natural sciences in the context of anatomy and physiology. Modern trends in medical thinking are characterized by empiricism, rationalism and realism (Wulff et al. 1993, 53–70). Including disease within the framework of scientific paradigms is a relatively recent phenomenon as until the eighteenth century intuitive beliefs, superstitions and the cult of authority predominated in Europe (Porter and Vigarello 2015, 307).

The research on various relations between the old Polish literature and medicine is preliminary. So far, there has not been any scientific monograph which would discuss the subject exhaustively (Krzysztofik 2015a; Gałaj-Dempniak 2010). The list of literary texts of ancient epochs which, in varying degrees, address medical subjects is extensive. Literary works, including aphorisms, maxims, proverbs, fables, epigrams, dialogues, memoirs, elegies, hagiographies, hymns, sermons, letters, morality plays, songs, satire and laments, spoke about medical treatments, the ill, medicines, doctors, barbers, health and disease. This issue is also present in everyday texts, i.e. guides, herbariums, guidelines, instructions of municipal and church authorities, astrological predictions, calendars, family diaries, etc. Studying the relations between the old Polish culture and medicine those various works of literature can be classified according to many criteria—e.g. literary genres (fiction, popular literature, everyday literature), degree of the subject’s presence (disease and treatment as the main subject or a secondary one, mentioned casually while speaking about more important problems), the nature of discussed diseases as well as the specific character of discourse related to health and disease.

Regardless of the classification criteria, both in fiction and popular literature of ancient epochs, the same standards of thinking about health and disease are present. They are witness to the dynamics of blending of discourses of different phenomenological orders. The issue is addressed in religious, medical, astrological, social, political, economic, folk, middle-class or popular discourses. Reading the old Polish literature makes us realize that religious, medical–astrological and social discourses are the most important and the most common mental ways of explaining the sense of suffering recorded in numerous pieces of writing. Individual works of literature can be dominated by one of them, however, the discourses may also blend and complement each other to a varying degree as eclectic thinking, inherent in the old Polish culture, does not make them mutually exclusive. The purpose of the present paper is to answer the following research questions:

1. What are the basic paradigms of thinking related to the above-mentioned discourses?
2. What are the relations among them?

Disease is an ambiguous term, therefore, in order to reach for clarity, the definition will be limited to the somatic sphere of man. I accept the assumption that disease is “an objectively determined […] state of the damage to the structure and function of a given body organ disproportionately to age” (Kuchowicz 1985, 101). The problem of the disease of the soul is not developed here as it will require a more extensive description in the form of a separate paper; however, it is mentioned in the context of the religious discourse.

## Religious Discourse

The foundation of the religious discourse in the old Polish literature is the authority of the Christian religion, the Bible and the magisterium of the Church. Disease gains a theological-moral dimension, based on the belief in a close relation between the natural and the divine orders.

The basis for thinking within the religious paradigm is theology presenting God as the omnipotent Creator, freely fixing and suspending the laws of nature. His action is always purposeful and meaningful (as demonstrated in the famous maxim *Deus et natura nil faciunt frustra*—God and nature do not work together in vain), however, at the same time, it remains mysterious and impenetrable by the human mind. Man as a creature brought into being, in life and in death, depends on his Creator completely. Disease and health are thus subject to the will of God who has the power of changing the course of events in a miraculous way (miracle as a suspension or change of the laws of nature). An explanation of the sense of disease in the old Polish religious discourse does not consist in finding physical or biological causes of it, but rather means referring to the mysterious, divine will. Medieval thinkers believed that:

God sends sickness to man to punish or to try him. Five ways of interpreting the occurrence of diseases were distinguished: 1. Among the righteous, diseases occurred to increase their merits through demonstrating patience. 2. To guard virtue against pride. 3. To break down the sinner. 4. To express the glory of God due to miraculous healing. 5. To initiate eternal punishment that God often sends during one’s lifetime (Pollak 1970, 190–191).

According to such assumptions, disease may equally be a sign of punishment for sins or a symbol of being chosen by God, which places it in the space of mystery and at the end of the sacred and profane axis. The man’s attitude towards physical suffering understood in this way is characterized by either uncertainty or confidence connected with waiting for God’s will as well as the lack of need to refer to a medical authority. The patient may rebel against the judgements of the Creator, expecting some kind of miraculous recovery or he may meekly come to terms with suffering and death. Christ, described in the Gospel as the one who heals both human body and soul, is the only and the most perfect doctor able to cure all ailments and even resurrect the dead. Under certain conditions a priest, acting as a servant of Christ, may also become a doctor.

This model of discourse in traditional old Polish literature constitutes the main and the most common way of explaining the sense of suffering. Examples of it present in various literary works of ancient epochs are numerous. Such a discourse can be found in sermons (particularly funeral sermons), theological treatises, devotional texts, meditation literature, religious guides as well as in fiction (both poetry and prose). Due to the lack of space, I shall present only a few examples.

Mikołaj Rej in *Żywot człowieka poczciwego* [The Life of an Honest Man, ed. 1568] preaches noblemen that man suffers voluntarily and at his own request, shortening his life by committing grave sins (Rej 1956, 496). For the author, disease is an apparent consequence of sin whereas health is an expression of life filled with devotion. A direct relation between religious attitude and diseases is also typical of the most famous diarist of the seventieth century, Jan Chryzostom Pasek, who sees good health (a recovery from typhoid during his journey to Denmark) as God and Mary’s interference in his life. At the same time, his recovery from a serious illness caused by binge-drinking is attributed to St. Anthony’s intercession (Pasek 1968, 89). A famous Dominican theologian, Mikołaj of Mościska, explains the causes of disease within the same religious paradigm. In his guide for the ill, he writes that all physical conditions come from divine providence (Mikołaj z Mościsk 1626). The monk believes that disease does not happen by accident. Instead, it makes the God’s plan present. Infallible Creator may keep everyone healthy and since, He does not do it then there must be some higher purpose. Understanding the divine will is inaccessible to human experience, and therefore, a hidden cause of physical suffering remains a mystery that can only be explained in reference to the supernatural reality.

This type of discourse, assuming the blending of the natural order with the dive one, can also be found in old Polish remedies of “the soul and the body” (e.g. in *Recepta duszna i cielesna* [Remedy of soul and body] by Hieronim Powodowski 1577–1589), which assume the superiority of the spiritual element over the material one. Disease is caused by grave sins, such as, for example, idolatry or blasphemy. The advantage of the soul over the body leads to the belief that humility, repentance, conversion, confession, Holy Communion, prayer, fasting, almsgiving, pilgrimage, the intercession of the saints, sacramental life or atonement are the best remedies (Krzysztofik 2013, 162–175).

In the religious discourse, spiritual healing is far more important than physical health as it refers to the eschatological dimension of existence. The ultimate and the most significant goal of mortal man is not to live a healthy life but to achieve eternal salvation. The acceptance of this way of thinking in the Old Polish literature leads to the perception of illness either as a trial (biblical Job—a model of involuntary suffering—becomes an example to follow for writers and poets) or as grace leading to conversion and repentance. Understanding the sense of physical suffering as a special sign of being chosen by God, present in Christian monastic spirituality, is associated with the belief that thanks to illness one can reach the desired state of moral and spiritual perfection (Gelis 2015, 57–61). In this way, physical ailments are explained by many writers of the old Polish literature, e.g. seventeenth-century poet Wespazjan Kochowski. Such an attitude allows him to accept suffering in a humble way.

Soteriological (eschatological) dimension of the man’s existence means that experiencing multiple physical ailments becomes a unique opportunity of cleansing the soul from sins. This vision of disease is reflected in many traditional old Polish sermons, meditation literature and hagiographical literature. Humble acceptance of the destruction of the body constitutes a visible proof of mastering ones virtues, self-denial, controlling one’s nature, fear or disgust. Martyrs suffering in the name of faith as well as the Saviour dying on the cross become ideals for the man who aims at imitating suffering Christ (*imitatio Christi*). The outcome of such suffering is eternal salvation. At the same time, accepting suffering means accepting the afflictions of Christ being complemented in one’s body, according to St. Paul’s theology, who in his Letter to the Colossians writes: “ I am glad when I suffer for you in my body, for I am participating in the sufferings of Christ that continue for his body, the church” (Colossians 1:24). Physical suffering, a privilege to accept and the sign of a special gift of God’s providence, assumes the sacred value. It is the realization of the God’s mysterious plan which aims at the eternal salvation of man.

## Medical and Astrological Discourse

In the old Polish literature, disease also becomes part of the medical discourse, closely related in the former periods to the astrological one, due to the importance attributed to astrology (Garin 1992). The union of medicine and divination astrology is typical of this kind of discourse, and its authorities include ancient thinkers, Greek philosophy as well as medieval Arab scholars and modern physicians. In the first place, there is Hippocrates, the ‘father of medicine’, then Aristotle, Galen, Avicenna, Averroes, Albumasar, Guido Bonatti, Girolamo Mercuriale and Paracelsus. Physical suffering is explained through the cult of authorities, whose teachings are accepted without any reservations. Knowledge is not verified but rather accumulated and compiled. The ancient authority functions following the same laws as the medieval or Renaissance one.

Medical discourse looks for the causes of the disease not in the divine will but in the outside world, since it refers to the philosophy and the order of nature. Bodily ailments are perceived as a phenomenon that can be understood in a natural way, therefore, in the treatment of the disease, one must analyse physical evidence rather than the supernatural powers.

The foundation of medical discourse present in the old Polish literature is related to the theory of macro- and microcosm and associated with it humoural balance and birth theory (*genitur*). The man, i.e. the small world, reflects all the elements of the great world. The body consists of humors and their balance guarantees health. Macro- and microcosm are connected by a series of interactions. Constantly changing planetary systems directly affect human health. The association between vegetative and sensual spheres of man and the world situated over the moon is conditioned by the time of birth. The arrangement of planets at the time of birth determines our susceptibility to specific diseases, thus determining the biological condition of the body. This concept is promoted in individual horoscopes. The importance of astrology in the old Polish medicine is associated with the concept of *homo zodiacus, homo signorum* (the zodiac man) which is a symbolic division of the human body into twelve areas corresponding to the signs of the zodiac.

Pathological states of the human body are explained by both biological and astral premises. Disease is caused by the imbalance of body fluids and the unfavourable system of planes. The patient can be cured by means of blood-letting, purging the body, cupping therapy, baths, medications and adherence to the principle of moderation in eating and drinking. Medical treatments are effective only when they are performed during favourable planetary radiations. The physician is the man who possesses the secrets of medicine and astrology. He is required to have the knowledge of medicines and proficiency in the observation of the movement of planets and stars (Słowakowic 1672). Strong influence of astral medicine in the collective mentality is observed until the end of the seventeenth century.

The examples of the medical–astrological discourse in the old Polish literature are numerous and developed mostly in functional texts. It appears in medical handbooks, descriptions of medical compositions or first-aid kits for travellers. The mentality which put medicine and astrology on equal footing is also visible in the sixteenth-century herbariums (written by Stefan Falimirz, Hieronim Siczyński or Marcin Siennik) where in addition to the lists of drugs or descriptions of effects of herbs there is also information on the influence of stars on the human body (Krzysztofik 2015b). The medical–astral discourse in the collective consciousness is well established in the old Polish astrological predictions, which were a very popular product of literature at that time. In the forecast for the years 1564–1584, Stanislaw of Rawa, basing on the observations of stars, predicts the coming of a number of diseases, especially the plague which arouses panic, and a broken leg of king Władysław Jagielło (the event of 1424) is explained referring to a negative effect of the solar eclipse. Applying the theory of birth, he also presents the list of diseases people will suffer from depending on their date of birth (Stanisław z Rawy 1565). The aetiology of disease as a result of the negative influence of planetary radiation is also presented in Old Polish calendars, which included a regular section on ‘the disease and the air’(Krzysztofik 2010, 366–370). Here, the typical cult of authority is combined with the atmosphere of horror, unclear predictions, a long list of possible physical ailments, the best time of administering medicines as well as specific advice related to medical treatments. Medicine is combined on equal footing with astrology and superstitions.

Despite the fact that the astral discourse was criticized in the old Polish period, its revaluation took place only with the advent of rationalism and empiricism of the Enlightenment.

## Social Discourse

The disease as a phenomenon examined within a universal dimension, referring to all social layers and groups, is inscribed into the old Polish social discourse, complementary with other discourses. Disease is seen as a huge, destructive force, which can have disastrous consequences as it threatens the existence of the entire nation and the state disrupting social hierarchy, demographic structure, economic model as well as the model of material and spiritual culture (Karpiński 2000).

The social discourse refers mostly to infectious diseases, commonly called ‘plague’, which constituted a challenge for medicine of those times and generated mass fear and psychosis. The most dangerous diseases included bubonic plague and pulmonary embolism. People also feared typhus, bacillary dysentery, malaria and smallpox (Kuchowicz 1972, 18). The huge scale of the phenomenon is testified by the fact that during the pandemic of bubonic plague in Europe in the years 1348–1350, 25 million people died, which was about 1/3 of the entire population.

A written testimony of the social discourse includes mostly old Polish functional texts, i.e. medical treatises, regulations, universals, instructions, regulations of municipal, church and royal authorities, municipal and hospital logs, preaching texts and guides describing ways of protection against ‘plague’. Their authors were mainly scientists and doctors as well as clergymen (e.g. Piotr Umiastowski, *Nauka o morowym powietrzu…* Kraków 1591; Sebastian Petrycy z Pilzna, *Instrukcyja abo nauka, jak się sprawować czasu moru* Kraków 1613; Stanisław Słowakowic, *Ratunek potrzebny podczas morowego powietrza…,* Kraków 1682, Hieronim Powodowski, *Recepta duszna i cielesna przeciw powietrzu morowemu*…, Poznań 1577–1589).

Regardless of the adopted aetiology of suffering (the will of God, natural reasons, astral radiations, etc.), common features of the social discourse are an attempt to tame the panic fear of pandemic and care of human population. Scientists, doctors and clergymen, aware of the horror of the situation, show the readers the way of salvation. They give specific medical, preventive and organizational advice. They describe the signs heralding the ‘plague’, discuss the system of coordinating activities of various groups of people during the epidemic, describe characteristic symptoms and the course of disease processes and advise what to do and what to avoid for the common good. The assessment of these recommendations from today’s point of view shows the helplessness of man against the disease perceived as a dangerous social phenomenon, upsetting the existing rhythm of life, and even ruining the existing law and order of the world.

The topic of the pandemic of bubonic plaque was also present in other types of discourse of the old Polish culture, for instance, in the religious discourse which included sermons and devotional literature (poetic pleading prayers to God asking Him for salvation from the plaque).

## Prioritizing the Discourses and Their Interference. Conclusions

A common feature of the three types of discourse on the disease discussed in the present paper is the lack of need for empirical verification, associated with the understanding of the constancy of knowledge and the cult of authority. The requirement to revalue mental paradigms comes only with the rationalism of the Enlightenment.

Within one type of literary product, a specific discourse may exist independently, in isolation from other types; however, the analysis of the old Polish literature leads to the realization that frequently mental models of explaining pathological states of the body permeate each other and enter into a variety of relations. In many texts, both of fiction and popular literature, the interference of discourses is observed. For example, the religious discourse characteristic of the work *Żywot człowieka poczciwego* by Mikołaj Rej mingles with the astral one. Calendars, astrological predictions (annual and perennial), herbariums, ‘recipes for the soul and the body’ include medical–astrological, religious and social discourses. In addition to medical and astrological interpretations of conditions, there appear prayers to God as the ultimate appellate as well as specific health advice and guidance referring to the organization of society during ‘bubonic plague’.

The union of several discourses, present in different phenomenological orders, taking place within one product of literature, is dependent on the existence of cultural phenomenon of valorization and prioritization. Otherwise, the coexistence of opposites would be irreconcilable. The analysis of the old Polish texts indicates the presence of the following hierarchy: the most important is the religious discourse which governs the two other discourses. The medical–astral discourse, with its specific rules and orders (e.g. taking medications and performing medical procedures according to planetary radiation), functions properly when the plague epidemic does not decimate the population. At the time of ‘plague’, its principles, rules and laws are suspended. In an extreme situation, the map of the sky proves useless and irrelevant. During the pandemic, the social discourse has primacy over the astral one—we must act quickly, regardless of the planets’ favour (Słowakowic, a XVIII century doctor, and also expert astrologer, reaches for medical and religious arguments and resigns from the astral ones, because in his opinion in the case of a sudden illness, one should act immediately, even in spite of unfavourable planetary radiations).

The superiority of the religious discourse over the medico–astral one is confirmed in the popular maxim “astra regunt homines, sed Deus regit astra:”—the stars rule over people, but God is above the stars (Basista 1994, 47–54). When the predictions concerning the occurrence of specific diseases, especially epidemic diseases, included in calendars and other predictive texts failed, the explanation was that related to the will of God and the conversion of sinners, whose fervent prayer spared them from physical evil. The existence of astrology, deplored by the Church’s teaching, was possible in the Christian culture of Western Europe only when it was subordinated to God, who through systems of planets, comets or eclipses, sends people warning signs, calling them to repentance and conversion.

Valorization and prioritization which guarantee primacy to the religious discourse are also associated with the awareness of the inevitability of death and thinking about the eschatological dimension of existence. The belief in life after death gives meaning to the actions of people in extreme situations such as the fight against ‘plague’. For example, Hieronim Powodowski in the ‘recipe for the soul and the body’ recommended that during a pandemic priests should remain with the sick people, serving them with the ministry. The author is aware of the fact that, in this way, they expose themselves to death, but eternal salvation is more important than health and life. Medical arguments are ruled over by the religious ones.

The three discourses related to the disease presented in the paper show that the occurrence of bodily diseases in the old Polish literature was not amenable to a single, simple and easy explication, but was part of a series of ideological models coexisting with each other. Even if these models were incompatible with each other (e.g. disease is a divine punishment or a reward; God or an arrangement of stars and planets decide about pathological states of the body), there were valorization and hierarchy between them, which served a coherent vision of the world in axiological terms. All disease states were therefore explained within different phenomenological orders, which did not come into conflict with each other but created a relationship of dependency. Medical–astral and social conceptions of reality were subject to the superior religious one. The lack of reference to realism, empiricism and rationalism omnipresent in popular mentality changed only in the eighteen century.
